# Personal, social, and environmental correlates of physical activity and sport participation in an adolescent Turkish population

**DOI:** 10.1186/s43161-022-00070-2

**Published:** 2022-03-16

**Authors:** Eren Timurtaş, Halit Selçuk, Eda Çınar, İlkşan Demirbüken, Yaşar Sertbaş, Mine Gülden Polat

**Affiliations:** 1 Department of Physiotherapy and Rehabilitation, Faculty of Health Sciences, Marmara Unversity, İstanbul, Turkey; 2 Centre de recherche du CHUS/Département de Psychoéducation, Universite de Sherbrooke, Sherbrooke, QC Canada; 3 Department of Internal Medicine, Fatih Sultan Mehmet Education and Research Hospital, İstanbul, Turkey

**Keywords:** Adolescent, Correlates, Physical activity, Sports

## Abstract

**Background:**

Benefits of physical activity has been shown for adolescents; however, there is a decline trend in number of adolescents meeting current WHO recommendations. This trend underlines the importance of identifying factors associated with adolescents’ physical activity level (PAL) with considerations of regional and cultural differences to plan and implement effective policies. Therefore, the aim of this study was to determine personal, ecological, and social factors associated with PAL and sport participation in Turkish adolescents aged 11–14 years. A cross-sectional study was conducted by including 996 adolescents aged between 11 and 14 years from 39 secondary schools in İstanbul, Turkey. Logistic regression analyses performed to identify the significant personal (age, gender, sleep time, screen time, BMIz score, having siblings), ecological (presence of playground, type of school transportation), and social (family income, engaging a physical activity with family, and preferred activity at school breaks) predictors of PAL and sport participation.

**Results:**

Adolescents who were active during break time at school (*p* < 0.001), engaging a physical activity with family (*p* < 0.001), and did not have a sibling (*p* = 0.029) were more likely to be physically active. Adolescents behaved active during break time at school (*p* < 0.001), had a playground at home (*p* < 0.001), spending time with family for physical activity (*p* < 0.001), and did not have a sibling (*p* = 0.021) were more likely to participate in a sport activity.

**Conclusions:**

Predictors of PAL in this study indicates the need to promote active break time in school, increased physical activity time with family, and to design environmental policies to increase number of playgrounds.

**Supplementary Information:**

The online version contains supplementary material available at 10.1186/s43161-022-00070-2.

## Introduction

Physical activity (PA) habits and the continuity of individual’s engagement in PA are of great importance for not only health benefits but also individual’s wellbeing and mental health. World Health Organization (WHO) [1] has recommended PA for all age groups and previous studies has provided evidence for the benefits of higher physical activity level (PAL) [2, 3]. Particularly, in children and adolescents, higher levels of physical activity have been linked to reduced risks of severe health problems, optimal wellbeing, physical fitness, improved cognitive function and academic performance, reduced risk of anxiety/depression, and body growth and development [2]. Nevertheless, in a pooled analysis of cross-sectional studies including 1.6 million school-going adolescents globally, 81.0% of the adolescents did not meet the current PAL recommendations [4] announced by WHO. It has been shown that physical activity level declines by an average of approximately 4% per year after the age of six [5] which continue to decline as children progress through childhood to adolescence [6].

This trend was also evident in Turkish population. According to a national report published by the Turkish Ministry of Health, 71.9% of adolescents do not exercise regularly [7]. Similar studies also reported that Turkish adolescents have low level of PAL and high level of sedentary behaviors [8, 9] which further underlines the need to identify the factors leading to this phenomenon.

Previous studies conducted in different countries identified potential factors associated with PAL and physical activity behaviors of adolescents considering individual, interpersonal, and environmental influences [10]. A recent review has pointed out that several factors including age, gender, parent activity level, physical activity and sport participation in school, peer support, and socioeconomic status could affect the PAL of adolescents [11]. Moreover, PAL has been reported to be linked with screen time of adolescents [12], choice of school transportation [10], and condition of school environment [13]. Since regional and cultural differences is an important determinant associated with PAL [14], identifying the factors related with PAL and physical activity behavior of Turkish adolescents in order to plan and implement national public health policies is warranted.

Even though numerous studies investigated the determinants of PAL in adolescent on understanding sociodemographic and environmental factors, there were no studies investigating these determinants in Turkish adolescent population. Therefore, the aim of this study was to determine the individual, social, and environmental factors associated with PAL and sport participation—as an indicator of high level of PAL—in adolescents aged 11–14 years in Turkish population.

## Material and methods

### Study design

This study used a cross-sectional survey design to investigate the association of physical activity level and sport participation of adolescents with the personal, social, and ecological factors. After obtaining the necessary approvals from local governments and the ethical approval from Ethics Committee of Marmara University Faculty of Health Sciences (No: 239 Date: 19.12.2019), the study was conducted between February 2020 and March 2020. There were 39 secondary schools in Üsküdar district of Istanbul, and the Education Ministry provided a random selection of schools for data collection. To select the sample, the secondary schools in the district were listed via an electronic medium and 8 of these schools were randomly chosen. However, due to COVID-19 pandemic, data collection was only completed in 3 schools (including 996 participants) before the national shutdown.

### Participants

**Fig. 1:**
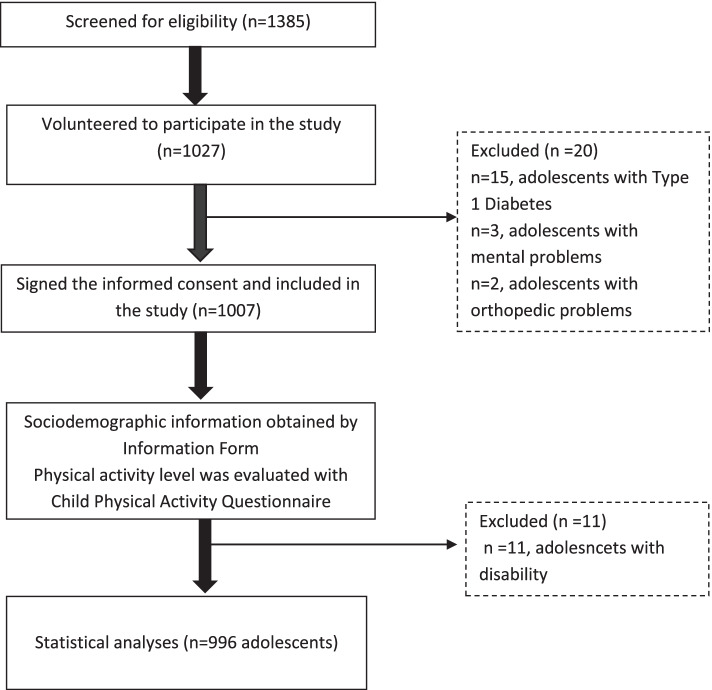
Flow chart of the study

Nine hundred ninety-six students aged 11–14 years were invited to take part in the study. Those who returned the informed consent form signed by their parents participated in the study. Students with orthopedic problems that prevent them from participating in physical activity, and those with any systemic, neurological, chronic diseases, or mental problems were not included in the study. The flow chart of the study was summarized in Fig. 1.

### Procedure

Data was collected in the classroom by making face-to-face interviews with each student, under the supervision of the teachers. Information Form (Additional file 1: Appendix 1) and Child Physical Activity Form were used as outcomes. Information was obtained from the administration and teachers on the screening day about students with special conditions (who have a disability, inclusive student, etc.), their answers were obtained, but they were excluded from the study.

### Outcomes

PAL and sport participation were measured by using Child Physical Activity Questionnaire (PAQ) and questioning sport-related habits of adolescents.

#### Child physical activity questionnaire

The questionnaire was developed to evaluate the physical activity level of primary schoolchildren aged 8–14, from the fourth grade to the eighth grade. The reliability and validity of the questionnaire have been well documented [15, 16]. The validity of the questionnaire in Turkish population was also reported in 2012 [17]. Each item of the questionnaire, except for the tenth question, which questions the disease status, is evaluated on a 5-point Likert scale and has an activity score between 1 and 5. “1” indicates low physical activity, “5” indicates high physical activity. The total score of the survey is 1–9. It is calculated by summing up the scores of the answers given to the question and dividing it by the number of questions. A cut-off point of 2.75 was used to identify adolescents who are active (a score of 2.75 or more) or inactive (a score of less than 2.75) [18].

#### Sport participation

The type of sport activity, duration, and frequency were questioned to decide regular sport participation. Adolescents were classified as regular sport participants if they involved in one of the previously identified sport activity at least once in a month [19] or not regular sport participants (i.e., those who did not involve in one of the previously identified sport activity at least once in a month).

### Predictors

#### Personal factors

Personal factors included age (continuous), gender (categorical: male or female), sleep time (continuous: calculated as hours spent sleeping on average per a day), and screen time (continuous: calculated as hours spent in front of the TV, computer, tablet or phone). BMI z score (BMIz) (continuous) was measured by adjusting weight for participants’ age and sex [20]. Siblings (categorical) was classified as “Yes” if the adolescent had at least one sibling; if they did not, it was classified as “No”.

#### Ecological factors

Playground (categorical) was classified as “Yes” or “No” depending on presence of park or playground in the adolescent’s neighborhood. Type of school transportation (categorical) were classified based on the type of transportation they use to get to school as “Physically active (e.g., walking, cycling)” or “Physically inactive (e.g., using bus).”

#### Social factors

Family income (categorical) was grouped into three categories as “Lower”/”Middle”/”Higher.” Family activity time (categorical) was classified as “Yes” or “No” depending on if adolescent spends time with family for physical activity. Adolescent preference for school breaks (categorical) was classified considering the type of activity that adolescents prefer at their break time as “active (e.g., playing tag)” or “inactive (e.g., sitting).”

### Data analysis

Descriptive statistics were used to summarize the demographic information of the participants, and all performance scores. The normality of data was visually evaluated by histograms, and Quantile–Quantile plots; and tested using the Shapiro–Wilk test. The observed outliers were removed from the data to improve the normality of the data. In the condition where data was not normally distributed after outlier removal, the log transformation was done for continuous variables.

Before the main analysis, the collinearity among independent variables were checked through variance inflation factor (VIF). Collinearity was determined to be present when the variance inflation factor was over 5 [21].

The two outcomes (physical activity level and sport participation) were regressed against 6 personal (age, gender, BMIz, sleep time, screen time, and siblings), 2 ecological (playground and school transportation choice), and 3 social (adolescent preference, family income, and family activity time) independent variables using logistic regression analysis. The binary variables (gender, adolescent preference, playground access, school transportation, sibling, family activity time) and ordinal variable (family income) were included into the regression analysis. The data was transformed into dummy variables with being female, being inactive, absence of a playground, using inactive mean of transportation, lower income, absence of a sibling, and lack of active time with family as the reference values.

The model was inspected visually for linearity, heteroscedasticity, and normality of the residuals, and goodness of the fit was evaluated using Hosmer-Lemeshow goodness of fit test, which is a Chi-square test conducted by dividing the sorted set into g=10 equal-sized groups [22]. Our previous study is consistent with the previous studies in the literature which showed that the inactivity rate in adolescents was around 80% in Turkish population [1, 2]. Based on this rate, a sample of at least 748 was needed to obtain 99% power with a confidence level of 95% and 5% Type 1 error, which is lesser than the current sample of 996 adolescents. All statistical analysis was done using R statistical software (Version 3.6.0, St. Louis, Missouri, USA), the package “ResourceSelection” [23]. The alpha level was .05.

## Results

Table 1 shows the characteristics of study participants stratified by gender and age. Of the 996 participants, 445 (44.7%) of them were female. Participants’ ages ranged from 11 to 14 years with a mean of 12.60 ± 1.10 years. Mean BMIz of the participants was 19.73 ± 3.52 kg/m^2^. Of 996 participants included 426 (42.8%) were active according to cut-off value of 2.75. The active participants were 276 (50.1%) for males and 150 (33.7%) for females. Mean PAQ score was 2.75 ± 0.71 for males and 2.49 ± 0.67 for females, which together with active percentage indicate lower PAL of female adolescents in this study.

**Table 1:** Characteristics of study participants

	Total (***n*** = 996)	11-year-old (***n*** = 219)	12-year-old (***n*** = 238)	13-year-old (***n*** = 270)	14-year-old (***n*** = 269)
**Gender (*n*, %)**					
**Males**	551 (55.3%)	111 (50.7%)	132 (55.5%)	155 (57.4%)	153 (56.9%)
**Females**	445 (44.7%)	108 (49.3%)	106 (44.5%)	115 (42.6%)	116 (43.1%)
**BMIz (mean, SD)**	19.73 ± 3.52	18.99 ± 3.25	19.31 ± 3.32	19.52 ± 3.37	20.87 ± 3.61
**Males**	20.17 ± 3.60	19.27 ± 3.28	19.74 ± 3.45	19.82 ± 3.06	21.54 ± 4.08
**Females**	19.17 ± 3.34	18.65 ± 3.21	18.78 ± 3.15	19.14 ± 3.76	20.03 ± 3.03
**Sleep time (mean, SD)**	506.75 ± 73.42	532.36 ± 72.19	521.35 ± 75.52	499.06 ± 71.45	480.81 ± 63.78
**Males**	504.46 ± 75.14	527.84 ± 73.74	517.97 ± 80.24	494.74 ± 75.70	486.21 ± 64.68
**Females**	509.61 ± 71.20	537.95 ± 70.27	525.53 ± 69.67	504.42 ± 66.18	474.12 ± 62.67
**Sitting time (mean, SD)**	544.03 ± 129.55	536.61 ± 109.45	531.98 ± 116.09	550.30 ± 134.78	556.50 ± 143.88
**Males**	539.11 ± 136.05	547.21 ± 105.92	529.31 ± 112.85	537.40 ± 143.18	543.27 ± 163.99
**Females**	550.18 ± 120.82	523.49 ± 113.82	535.28 ± 120.10	566.28 ± 124.37	572.89 ± 118.97
**Screen time (mean, SD)**	132.88 ± 98.08	108.84 ± 81.46	124.50 ± 92.88	151.94 ± 109.92	139.97 ± 94.16
**Males**	139.82 ± 98.52	113.17 ± 68.65	128.70 ± 101.53	164.52 ± 114.31	143.48 ± 90.59
**Females**	124.16 ± 96.94	103.48 ± 97.33	119.31 ± 82.17	136.37 ± 104.48	135.63 ± 98.57
**Preference for school break**^**a**^ **(Active) (*n*, %)**	669 (67.2%)	177 (80.8%)	176 (73.9%)	168 (62.2%)	148 (55.0%)
**Males**	394 (71.5%)	98 (88.3%)	106 (80.9%)	98 (63.2%)	92 (59.7%)
**Females**	275 (61.8%)	79 (73.8%)	70 (65.4%)	70 (60.9%)	56 (48.3%)
**Playground**^**b**^ **(Yes)**	766 (76.9%)	171 (78.1%)	177 (74.4%)	210 (77.7%)	208 (77.3%)
**Males**	423 (76.8%)	88 (79.3%)	96 (73.3%)	123 (79.4%)	116 (75.3%)
**Females**	343 (77.1%)	83 (77.6%)	81 (75.7%)	87 (75.7%)	92 (79.3%)
**School Transportation**^**c**^ **(Active) (*n*, %)**	318 (31.9%)	52 (23.7%)	68 (28.6%)	86 (31.9%)	112 (41.6%)
**Males**	184 (33.4%)	25 (22.5%)	42 (32.1%)	50 (32.3%)	67 (43.5%)
**Females**	134 (30.1%)	27 (25.2%)	26 (24.3%)	36 (31.3%)	45 (38.8%)
**Siblings**^**d**^ **(Yes) (*n*, %)**	847 (85.1%)	184 (84.4%)	203 (85.3%)	223 (82.6%)	237 (88.1%)
**Males**	474 (86.0%)	94 (84.7%)	112 (85.5%)	132 (85.2%)	136 (88.3%)
**Females**	373 (83.8%)	90 (84.1%)	91 (85.0%)	91 (79.1%)	101 (87.1%)
**PAQ Score (mean, SD)**	2.64 ± 0.70	2.78 ± 0.66	2.81 ± 0.71	2.59 ± 0.68	2.41 ± 0.69
**Males**	2.75 ± 0.71	2.84 ± 0.70	2.96 ± 0.68	2.65 ± 0.70	2.61 ± 0.70
**Females**	2.49 ± 0.67	2.71 ± 0.62	2.64 ± 0.70	2.51 ± 0.64	2.14 ± 0.57
**PAQ classification**^**e**^ **(Active) (*n*, %)**	426 (42.8%)	114 (52.1%)	129 (54.2%)	107 (39.6%)	76 (28.3%)
**Males**	276 (50.1%)	63 (56.8%)	88 (66.7%)	65 (41.9%)	60 (39.2%)
**Females**	150 (33.7%)	51 (47.2%)	41 (38.7%)	42 (36.5%)	16 (13.8%)
**Sport Participation**^**f**^ **(Yes) (*n*, %)**	603 (60.5%)	128 (58.7%)	152 (63.9%)	164 (60.7%)	159 (58.9%)
**Males**	371 (67.3%)	81 (69.2%)	89 (71.2%)	109 (66.5%)	92 (63.4%)
**Females**	232 (52.1%)	47 (46.5%)	63 (55.8%)	55 (51.9%)	67 (53.6%)

^a^Shows the percentage of adolescents preferring an active break time activity

^b^Shows the percentage of adolescents preferring an active break time activity

^c^Shows the percentage of adolescents who have a playground in neighborhood

^d^Shows the percentage of adolescents who have at least one sibling

^e^Shows the percentage of active adolescents calculated from PAQ score with a cut-off value of 2.75

^f^Shows the percentage of adolescents who participated in a sport activity

### Physical activity level

The analysis showed the suggested model, yielding the χ^2^ (Chi-square) of 3.29, was fit the data well (*p* = 0.91). There was a significant relationship between the PAL and sleep time, preference for break activity, having a sibling, and engaging a physical activity with family of adolescents (*p* < 0.05). Adolescents who were active during break time at school (OR = 4.28, *p* ≤ 0.001), spending less time for sleep (OR = 2.61, *p* = 0.042), engaging a physical activity with family (OR = 1.21, *p* ≤ 0.001) and who did not have a sibling (OR = 6.51, *p* = 0.029) were more likely to be physically active, respectively (Table 2).

**Table 2:** Association between the PAQ score of adolescents and the predictors

	Estimate	Std. Error	Z	*p*	OR
Age*	0.10	0.06	1.58	0.114	1.10
Gender (male)	0.27	0.14	1.86	0.063	1.31
BMIz*	0.60	0.65	0.92	0.359	1.82
Sleep time *	0.96	0.47	2.03	0.042*	2.61
Screen time*	0.05	0.09	0.60	0.550	1.05
Preference for school break (active)	1.45	0.17	8.79	< 0.001*	4.28
Playground (yes)	0.28	0.17	1.64	0.100	1.32
School transportation (physically active)	0.15	0.15	1.00	0.316	1.16
Family income (higher)	0.09	0.20	0.45	0.652	1.09
Siblings (yes)	− 0.43	0.20	− 2.18	0.029*	6.51
Family activity time (yes)	0.19	0.05	3.72	< 0.001*	1.21

*Continuous variable

AIC: 1204.3

Goodness of fit test

χ^2^ (Chi-square) = 3.29, df (g-2) = 8, *p* value = 0.914

### Sport participation

The suggested model, yielding the χ^2^ of 5.87, fit the data well (*p* = 0.661). The sport participation was significantly associated with preference for break activity, availability of playground, having a sibling, and engaging a physical activity with family (*p* < 0.05). Adolescents who are active during break time at school (OR = 2.35, *p* ≤ 0.001), had access to playground (OR = 1.75, *p* ≤ 0.001), reported some level of activity with family (OR = 1.23, *p* 0.021), and who did not have a sibling (OR = 0.62, *p* = 0.021) were more likely to participate in a sport activity (Table 3).

**Table 3:** Association between the sport participation of adolescents and the predictors

	Estimate	Std. error	Z	*p*	OR
Age*	− 0.02	0.06	− 0.30	0.767	0.98
Gender (male)	0.23	0.14	1.63	0.104	1.26
BMIz*	− 0.42	0.65	− 0.64	0.519	0.66
Sleep time *	0.23	0.44	0.52	0.606	1.25
Screen time*	− 0.05	0.08	− 0.60	0.550	0.95
Preference for school break (active)	0.85	0.15	5.88	< 0.001*	2.35
Playground (yes)	0.56	0.16	3.45	< 0.001*	1.75
School transportation (physically active)	− 0.03	0.15	− 0.18	0.856	0.97
Family income (higher)	0.04	0.20	0.21	0.833	1.04
Siblings (yes)	− 0.47	0.20	− 2.31	0.021*	0.62
Family activity time (yes)	0.20	0.05	4.16	< 0.001*	1.23

*Continuous variable, AIC: 1248

Goodness of fit test

χ^2^ (Chi-square) = 5.873, df(g-2) = 8, *p* value = 0.661

## Discussion

This study examined the associations between personal, ecological, and social factors and adolescents’ physical activity level and sport participation in a sample of Turkish population. We found evidence that being active during break time at school, spending less time for sleep, engaging a physical activity with family, and not having a sibling were associated with being physically active in adolescents. Similarly, being active during break time at school, having a playground at home, engaging a physical activity with family, and not having a sibling were associated with participating in sports.

Of the six estimated personal factors (age, gender, sleep time, screen time, BMIz, and siblings), only spending less time in sleeping and not having a sibling were associated with high level of PAL of adolescents. The relationship between sleeping time and PAL has been reported globally [24]. For example, studies done in adolescents in the Europe and North America consistently reported a significant link between less sleep time and higher level of participation in PA and sport [25, 26]. Pedisic et al. [24] also reported that spending more time in sleep is not only associated with low level of PA but also sedentary behavior of children and adolescents. On the other hand, there are other studies that reporting the opposite, where lower sleep duration was related to sedentary behavior of children [27, 28]. This might be related to the differences in optimum duration of sleep time determined in different studies. Contrary to the literature [29], having a sibling was negatively associated with PAL. Gender itself was also associated with PAL; males had higher PAL compared to females in the previous study [30]. In this study, however, even though the difference between genders were observable in the descriptive data, we did not found gender as a predictor neither of PAL nor sport participation. Similarly, age and BMI were not a predictor of PAL or sport participation in this study; yet previous studies including adolescents in different age groups indicated a decline in PAL with age [30]. For example, adolescents aged 10- to 14-year-old had higher PAL compared to those aged 15- to19-year-old [30]. In our study, we only included adolescents 10- to 14-year-old which did not allow us the track the changes in PAL through adolescence. Surprisingly, this study did not found a relationship between screen time and PAL, which was observed in the previous studies where screen time was linked to sedentary behaviors [31].

It has been indicated that the environmental factors are less relevant to PAL compared to social and parental factors [32]; yet the presence of playground in the neighborhood was significantly associated with sport participation in this study but not with PAL. The presence of playground and its association with PAL was investigated from different aspect in a previous study. The study reported that there was a difference in how a place was perceived as a playground by parent. Adolescents whose parents thought they had a playground, even if they had the same environmental facilities with others, were more active than those whose parents perceived that they didn't have a playground [33]. On the other hand, physical environment and having an accessible field is crucial for playing or practicing sports [34]. Our participants revealed that having a playground opportunity lead them to participate in a sport regularly.

Active transportation to school has been also shown to be associated with higher PAL and sport participation in many studies [35]. However, we did not observe this relationship in this study. This may be due to the fact that only a small percentage of our study sample was using active transportation to get to school and Turkish adolescence rarely uses bicycles as a means of transportation compared to other countries (e.g., the Netherlands) [36].

The importance of peer support on participating in sports and PAL has been reported previously [37]. Our survey did not question about the relationship among peers and how they support each other, yet participants were questioned about how they are spending their time during their break time. Those who preferred spending time with their peers in an activity making them physically active, such as playing tag, had higher PAL levels. This tendency has been also reported in the current literature of qualitative and quantitative studies [11, 38]. Similarly, children spending some time with their family for any sort of PA had higher PAL in both current study and previous studies [39]. Family income was another social factor which was deemed to be linked with PAL of adolescents in the previous studies [38]. For example, in a study done in the USA, high family income was associated with the increased level of moderate to vigorous physical activity in adolescents [40], yet this relationship was not significant in a sample of Turkish population.

This study also presented with some limitations. Firstly, due to inability to include adolescents from different cities and regions of Turkey, the results cannot be generalized to all Turkish population. Secondly, we used a questionnaire (PAQ) to measure the PAL of adolescents and did not use a device-based (e.g., accelerometer) or performance-based (e.g., shuttle run test) measurement methods. Thirdly, we were unable to include all predictors specially to investigate the socioeconomic and cultural predictors of PAL and sport participation. Therefore, it is recommended that future studies should include adolescents from different regions and cities of Turkey and should further investigate the relationship between socioeconomic and cultural variables and PAL since these factors may cause lower PAL among adolescents through reduced access. Also, future research could combine the factors identified in this study with previous physical activity interventions to enhance these interventions.

## Conclusions

In conclusion, this study showed that half of the male adolescents and more than 65% of the female adolescents were inactive, which underlines the need for implementing physical activity policies for these age group. Policies focusing on adolescents of Turkish population should consider the predictors in this study when implementing physical activity guidelines. The identified factors related to PAL in this study indicates the need to promote active break time in school, adolescents’ physical activity time with family, and optimizing sleep time. Also, it is important to design environmental policies to increase and optimize the playgrounds.

## Supplementary Information

**Additional file 1.**

## Data Availability

Not applicable.
