# Supplementary material for: Personal, social, and environmental correlates of physical activity and sport participation in an adolescent Turkish population
Source: Bull Fac Phys Ther. 2022 Mar 16;27(1):11. doi: 10.1186/s43161-022-00070-2 (PMC8923827; doi:10.1186/s43161-022-00070-2)
Supplement: Supplementary file 1 — Additional file 1. [file 43161_2022_70_MOESM1_ESM.docx]

**Information Form**

**School Name: Grade:**

1. Date of Birth:
2. Gender: Female Male
3. Height:
4. Weight:
5. Place of Birth:

| 7. Where did you grew-up: Village | Town | City |
| --- | --- | --- |
| 8. Do you have any chronic disease? |  |  |

1. Have many siblings do you have?
2. Where do you live :
   1. Apartment
   2. Site Does the site have a playground: Yes No
   3. Other (Please specify)
3. Are there any parks/playgrounds in your neighboorhood? Yes No
4. Are you involved in any kind of sport/physical activity? Yes No
5. The type of the sport/physical activity:
6. Since when are you continuing this activity (months, years):
7. Are you doing this activity regularly? Yes No
8. Once in a week 2-4 times per week 5-6 times per week Everyday 1-2 times per month 3-4 times per month
9. How long does this activity takes?
10. Where do you do this activity? School Home Park/Playground Sport center Other (Please specify)
11. Does any of your family members involved in a physical activity/sportsexercise? Yes No
12. If yes, Please specify the relative and type of activity:
13. Do you attend to physical activity/sports with your family? Yes No
14. What type of activity? Hiking/Jogging Cycling Playing games in park/playground Football/Basketball/Voleyball,etc. Swimming
15. How frequently do you do this this activity?
16. Once in a week 2-4 times per week 5-6 times per week Everyday 1-2 times per month 3-4 times per month Rarely
17. How long does this activity takes?
18. On average, how many hours do you sleep per day?
19. On average, how many hours do you spend sitting per day?
20. On average, how many hours do you spend in front of TV/Tablet/Phone/Computer per day?
21. How many hours do you spend in school per day?
22. What do you prefer to do in breaktimes at school? Sitting in class Walking around the schoolyard Skipping Rope Table tennis Playing chess Attending club activity Other
23. How do you travel to school? By bus By car By schhol bus By bicycle Other
24. How long is it take to travel to school?
25. Education level of your mother? Illiterate Literate Primary School Secondary School High School Higher Education
26. Education level of your father? Illiterate Literate Primary School Secondary School High School Higher Education
27. Occupation of your mother:
28. Occupation of your father:
29. Average income of your family per month:
30. Do you have a physical education lesson in school? Yes No
31. Does your school has a sport center? Yes No
32. Does your school has a schoolyard? Yes No
33. What type of activity do you do in physical education lesson? Running Football Basketball Voleyball Other
